# The contemporary management of prostate cancer

**DOI:** 10.3322/caac.70020

**Published:** 2025-06-26

**Authors:** Deep Chakrabarti, Peter Albertsen, Aidan Adkins, Amar Kishan, Vedang Murthy, Chris Parker, Angela Pathmanathan, Alison Reid, Oliver Sartor, Nicholas Van As, Jochen Walz, Alison Tree

**Affiliations:** ^1^ Uro‐Oncology Unit The Royal Marsden National Health Service Foundation Trust Sutton and London UK; ^2^ UConn Health Farmington Connecticut USA; ^3^ Europa Uomo Antwerp Belgium; ^4^ Ronald Reagan UCLA Medical Center University of California‐Los Angeles Medical Center Santa Monica California USA; ^5^ Tata Memorial Hospital and Advanced Center for Treatment Research and Education in Cancer Homi Bhabha National Institute Mumbai India; ^6^ The Institute of Cancer Research London UK; ^7^ Mayo Clinic Comprehensive Cancer Center Mayo Clinic Rochester Minnesota USA; ^8^ Institut Paoli‐Calmettes Cancer Center Marseille France

**Keywords:** medical oncology, prostate neoplasms, radiation oncology, survivorship, urology

## Abstract

Prostate cancer is the most common cancer in two thirds of the world, with an expected doubling in both incidence and mortality in the next two decades. No strong environmental associations exist for the development of prostate cancer; therefore, lifestyle measures are unlikely to mitigate this increasing burden. The last three decades have seen rapid developments in the diagnostic and therapeutic landscape of prostate cancer, including multiparametric magnetic resonance imaging, positron emission tomography, robotic surgery, image‐guided hypofractionated and stereotactic radiotherapy, novel anti‐androgens and radioligand therapies. Prostate cancer is unique in that not everyone with a diagnosis needs treatment, and active surveillance is the preferred option for some. This review discusses the contemporary management of all stages of prostate cancer in the light of these modern developments, enabling holistic individualization of treatment, and describes the promise of future research to further improve outcomes.

## EPIDEMIOLOGY

### Incidence and mortality

An estimated 1.5 million individuals are diagnosed with prostate cancer and almost 400,000 die from it worldwide each year. Prostate cancer is the most commonly diagnosed cancer in two thirds of the world’s countries and has a positive correlation with a country’s wealth as measured by the human development index. The highest age‐standardized incidence rates (per 100,000 males) are observed in Northern Europe (82.8), Australia/New Zealand (78.1), the Caribbean (73.8), and North America (73.5), whereas regions in Asia and Africa have the lowest overall rates but the highest annual increases.^1^, ^2^, ^3^ Prostate cancer mortality rates do not correlate with incidence rates most likely because of disparities in screening, diagnosis, and treatment between wealthy and poorer countries. Prostate cancer is the leading cause of cancer death in men in 52 countries, including countries in the Caribbean, sub‐Saharan Africa, Central and South America, and Sweden in Europe.^1^ Wealthier countries in Asia, such as Japan and South Korea, have a lower incidence than Western countries with a similar human development index.^4^ Worldwide, the number of new cases of prostate cancer will likely double from 1.4 million in 2020 to 2.9 million in 2040, and the annual number of deaths will increase from 375,000 in 2020 to approximately 700,000 by 2040.^5^

### Risk factors

Few known environmental or lifestyle factors have a concrete association with prostate cancer. The only established risk factors are advancing age, African/Caribbean descent, a positive family history, and certain genetic mutations. More than 70% of all prostate cancers are diagnosed in individuals older than 65 years.^6^, ^7^

Smoking, being overweight or tall, consuming higher quantities of dairy products and calcium or diets with low levels of vitamin E or selenium may increase the risk of prostate cancer.^8^, ^9^, ^10^, ^11^, ^12^ Gut microbiomes may also play a role in the development of castration resistance by the modulation of signaling pathways by dietary polyunsaturated fatty acids and metabolism of dihydrotestosterone and testosterone.^13^, ^14^, ^15^ Specifically, commensal gut micriobiota can convert androgen precursors into active androgens and thereby provide an alternative source of androgens, which confer endocrine resistance.^16^

The increased incidence in the Caribbean countries or in countries of sub‐Saharan Africa likely reflects increased genetic susceptibility in those of West African descent.^17^, ^18^ Those with low testosterone have a reduced risk, whereas testosterone‐replacement therapy does not appear to increase the risk of developing prostate cancer.^19^, ^20^ Ejaculation frequency during adulthood (≥21 compared with four to seven ejaculations per month) may reduce the chance of a prostate cancer diagnosis.^21^ Unfortunately, there are no strong modifiable risk factors for this disease.

### Genetic predisposition

Prostate cancer correlates strongly with a family history of any cancer. Nine percent of individuals with prostate cancer have a family history of cancer and present six to seven years earlier than those with nonhereditary disease.^22^, ^23^, ^24^ Those who have a first‐degree relative with prostate cancer have a two‐fold increased risk of diagnosis. The risk is highest if a family member was diagnosed before the age of 60 years.^25^ If a man has more than one first‐degree with prostate cancer, his risk of diagnosis increases to more than three‐fold and depends on the relatives involved: father and a brother (relative risk [RR], 5.5), two brothers (RR, 7.7), three brothers (RR, 17.7).^22^, ^25^, ^26^ Prostate cancer is called familial if an individual has more than three affected relatives, three successively affected generations, or two affected relatives diagnosed before the age of 55 years.^27^

Germline (inherited) mutations confer an increased risk of developing prostate cancer and include the following genes: BRCA1, BRCA2, ATM, ATR, mismatch repair (MMR) genes (MSH2, MSH6, and PMS2), CHEK2, RAD51D, NBS1, and PALB2.^28^ The incidence of germline mutations mediating DNA repair is higher in those who have metastatic disease versus localized prostate cancer.^29^ Germline BRCA1/2 mutations are present in approximately 6% of patients with prostate cancer.^30^ The mutations that confer the highest risk of developing prostate cancer are those in BRCA2^29^, ^31^ (eight‐fold) and HOXB13^32^, ^33^, ^34^ (three‐fold).^33^, ^35^ More than 170 single nucleotide polymorphisms (SNPs) have been associated with prostate cancer, but their contribution to cancer development is poorly defined.^36^

## SCREENING

Prostate‐specific antigen (PSA) is a glycoprotein enzyme secreted by the epithelium of the prostate gland. PSA helps break down large proteins in semen, thereby decreasing seminal viscosity and improving sperm motility and fertility.^37^ Normally, only a small amount of PSA diffuses into the bloodstream. However, conditions that disrupt the prostate microarchitecture (i.e., trauma, prostatic inflammation, or malignancy) cause an increased diffusion of PSA into extracellular space and consequently into the bloodstream through lymphatic channels that can be detected by a serum assay. PSA levels may be increased in benign conditions like benign prostatic hyperplasia and prostatitis or after perineal trauma, ejaculation, and in cancer.^37^ Certain drugs may lower PSA levels, and these include thiazide diuretics, nonsteroidal anti‐inflammatory drugs, statins, and, more significantly, 5‐alpha‐reductase inhibitors.^37^

Theoretically, prostate cancer is a good target for screening because of high global mortality rates and the availability of a convenient blood test to measure PSA. The primary aim of PSA screening is to identify cancers earlier, when their natural history can be altered by effective treatments.^38^

However, PSA is an unreliable marker for prostate cancer; most individuals with elevated levels do not have prostate cancer, and a normal PSA test does not rule out having the disease.^39^ Compared to an unscreened population, a single PSA test does not improve mortality after 10 years and only marginally improves prostate cancer mortality after 15 years (0.09%). PSA testing does lead to an increased diagnosis of low‐risk prostate cancer cases.^40^, ^41^ The Prostate, Lung, Colorectal, and Ovarian (PLCO) Cancer Screening Trial (ClinicalTrials.gov identifiers NCT00002540, NCT01696968, NCT01696981, and NCT01696994), which recruited 76,693 men from 10 American centers identified no mortality benefit from screening with PSA and digital rectal examination (DRE). However almost all men in the control arm also received a PSA test, which reduced the trial's power to detect a clinically meaningful benefit.^42^, ^43^ A secondary analysis of the data established that a baseline PSA level could serve as a long‐term risk factor for clinically significant disease. Those with PSA levels <1 ng/mL likely need no further screening.^44^

The European Randomized Study of Screening for Prostate Cancer (ERSPC) identified a 20% reduction in prostate cancer mortality but also noted that 570 men needed to be screened and 18 men needed to be diagnosed to prevent one prostate cancer death, although these numbers were reduced with longer follow‐up.^45^, ^46^, ^47^, ^48^ Unfortunately, the risk of overdiagnosis was substantial. Forty percent of men had low‐risk disease identified that might never have become clinically apparent.^49^ The Rotterdam cohort of the ERSPC study demonstrated a reduction in progression to metastatic disease by 24% and a reduction in prostate cancer mortality by 31% with screening. The Göteborg randomized trial also recorded a 29% reduction in prostate cancer mortality; however, 221 men needed to be invited and nine needed to be diagnosed to prevent one death from prostate cancer.^50^ A Cochrane meta‐analysis of older clinical trials failed to detect a clinically significant reduction in prostate cancer‐specific mortality.^38^

Prostate cancer screening based solely on PSA risks overdiagnosis and overtreatment of indolent disease, psychological effects, including anxiety or depression, and the potential complications associated with biopsy or overtreatment.^51^, ^52^ Therefore, although there is a possible reduction in prostate cancer mortality, the risks of overdiagnosis and overtreatment have deterred widespread adoption of screening. Several modifications, including models incorporating clinical variables, PSA dynamics, magnetic resonance imaging (MRI), risk calculators, and genetic markers (including SNPs) may lower these risks.^53^, ^54^, ^55^ A screening algorithm with PSA and MRI followed by MRI‐directed, targeted biopsy reduced the diagnosis of clinically indolent disease by half.^56^, ^57^

The European Union has now invited countries to pilot screening using PSA and MRI for risk assessment.^58^ The PRAISE‐U project encourages early detection and diagnosis of prostate cancer through customized and risk‐based screening programs.^59^ A Swedish cohort of such organized testing has been reported in which 35% of 68,060 invited men underwent PSA testing, and the combined approach of MRI and PSA density avoided a biopsy for >50% men with a PSA of ≥3 ng/mL.^60^

Individuals in families with hereditary cancer syndromes or known carrier mutations for BRCA1/2 may benefit from targeted screening.^61^, ^62^ Initial results from studies evaluating targeted screening based in men with higher genetic risk^63^, ^64^ have been reported.^63^, ^64^, ^65^, ^66^ Initial results of a mobile case‐finding project piloted in the United Kingdom could potentially lead to raising health awareness and address health inequalities.^67^ A large clinical trial from the United Kingdom is due to start recruiting in 2025 to compare the accuracy and cost‐effectiveness of potential screening methods, including PSA tests, fast MRI scans, or genetic tests for those at a higher risk.^68^

We do not recommend routine PSA screening of all individuals, and PSA testing should always be guided by an informed discussion between the health care professional and the individual on personalized risk (ethnicity, number of first‐degree relatives involved).^27^, ^69^, ^70^, ^71^, ^72^, ^73^, ^74^, ^75^, ^76^ MRI before biopsy is likely to play a prominent role in any future studies of screening. Risk calculators that incorporate clinical factors, MRI features, and blood or urinary biomarkers have been described to aid in the detection of clinically significant disease at biopsy but require wider multicentric external validation toward routine adoption in clinical practice.^77^, ^78^

## DIAGNOSIS

Most cases of early prostate cancer are asymptomatic. Many patients present with unrelated urologic issues that prompt a PSA test. These include urinary symptoms, such as frequency, passing urine at night, hesitancy, incomplete voiding,^79^ or sexual side effects like difficulties in achieving an erection.^80^

Advanced or metastatic disease can present with pain, typically back or bone pain. Very advanced disease can lead to metastatic spinal cord compression with radiating back or leg pain, leg weakness, numbness, tingling, paralysis, or incontinence.^81^

The risk of diagnosing a clinically significant prostate cancer depends on multiple factors: age, family history, PSA level, and, when advanced, a DRE. Risk calculators are available that incorporate these factors.^82^ The most common finding on DRE is the presence of a hard, fixed nodule. Other findings may include asymmetry or firmness. The presence of a stony, hard prostate on DRE signifies locally advanced disease. If an MRI is to be done, a DRE is not always necessary.^83^

A multiparametric MRI is recommended on clinical suspicion of prostate cancer, prior to a biopsy.^84^, ^85^, ^86^, ^87^ MRI‐directed biopsies are at least twice as accurate in identifying clinically relevant cancer compared with systematic transrectal biopsies alone. MRI also allows >25% of individuals to avoid subsequent biopsies that would likely diagnose insignificant disease.^84^, ^85^, ^88^ A suspicious MRI (≥3 according to the Prostate Imaging Reporting and Data System, PIRADS) should be followed by targeted and systematic biopsies. A biopsy can be omitted if the MRI is negative (PIRADS≤2) or with low clinical suspicion, if high‐quality MRI and expertise of reader are provided.^75^ MRI before biopsy is always recommended. Global adoption of this strategy with adequate quality control is potentially challenging.^89^ MRI provides details about local extent, seminal vesicle involvement, and extraprostatic disease and can help plan surgical techniques to increase negative margins, particularly with regards to neurovascular bundle sparing and excision of potential extraprostatic sites, but leads to stage migration.^90^, ^91^, ^92^

A histologic diagnosis of prostate cancer is made by assessing the loss of normal glandular architecture and disruption to the basal membrane, loss of surrounding basal cells, and nuclear atypia of luminal cells.^93^ The aggressiveness of an adenocarcinoma is reflected in the degree of differentiation on histopathology and is graded using the Gleason score (GS). Several changes have been made over the years, with modifications to grading and reporting.^94^, ^95^ The GS on biopsy is a sum of two numbers: the grade of the predominant pattern added to the grade of the highest grade pattern seen. Gleason grades range from 3 (moderately differentiated cancer cells) to 5 (no glandular features, sheets of abnormal cells). If only one Gleason grade is present within the biopsy, then it is doubled.^96^, ^97^, ^98^, ^99^ Overall grade groups (GGs) have been recommended since 2014 and are as follows: GG 1 (GS ≤ 6), GG 2 (GS 3 + 4 = 7), GG 3 (GS 4 + 3 = 7), GG 4 (GS 4 + 4 = 8), and GG 5 (GS 9–10).^100^ Commercially available tissue‐based biomarkers for risk stratification are now available (e.g., Prolaris [Myriad Genetics], Decipher [Veracyte, Inc.], Oncotype DX Prostate [Genomic Health], ProMark [Metamark Genetics Inc.]) and may help facilitate an individualized approach to treatment.^101^

Most prostate cancers originate from the epithelium and thus are carcinomas. Other rare histologies include sarcoma (derived from mesenchyme) and lymphoma.^102^ Pathologically, the most common type of prostate cancer is an acinar adenocarcinoma that originates from the prostatic secretory epithelium in the peripheral part of the gland and accounts for the majority of all newly diagnosed cases.^102^ Other histologic subtypes include ductal adenocarcinoma (3.2%, most mixed with acinar adenocarcinoma), preductal adenocarcinoma (0.4%–0.8%), neuroendocrine carcinomas (including small cell neuroendocrine carcinoma, 1%–5%; large cell neuroendocrine carcinoma; and treatment‐related neuroendocrine carcinoma), squamous carcinoma (<0.6%), adenosquamous carcinoma, or adenoid cystic carcinoma (basal cell carcinoma).^102^, ^103^, ^104^, ^105^ Adenosquamous carcinoma is commonly associated with prior treatment.^106^, ^107^, ^108^ Most variant pathologies have a poorer prognosis than acinar adenocarcinoma.^109^, ^110^ Treatment‐related neuroendocrine prostate carcinoma occurs because of transdifferentiation of a castration‐resistant prostate cancer (CRPC) after androgen‐deprivation therapy (ADT) and comprises 10%–15% of all CRPCs.^111^ Their prognosis is dismal and often less than one year.^112^ Focal neuroendocrine differentiation, however, may be a component of adenocarcinomas with a high Gleason grade.^102^

Disease risk stratification^76^, ^113^, ^114^, ^115^ is routinely adopted before the initiation of treatment and after surgery.^113^, ^114^, ^116^, ^117^, ^118^ Patients with low‐risk disease (T1/T2 tumor, GS ≤6, PSA ≤10 ng/mL) do not require further staging. Patients with intermediate‐risk disease require further staging investigations (MRI or computed tomography [CT] of the abdomen and pelvis and technetium‐99m bone scan), except those with GG 2 disease. For high‐risk disease, the cross‐sectional imaging should also include the thorax. Those with poor general health who may not be fit for any treatment and those who refuse treatment do not require further staging investigations.^75^, ^113^ Predictive nomograms and molecular biomarker tests (e.g., Prolaris, Decipher, Oncotype DX Prostate, or ProMark) improve risk stratification and can help predict local or distant recurrences after radical primary treatment.^119^, ^120^, ^121^, ^122^

Prostate‐specific membrane antigen (PSMA) is a transmembrane protein that is overexpressed on prostate cancer cells, although approximately 10% of all prostate cancers may be PSMA‐negative.^123^ More than 90% of intraprostatic lesions are PSMA‐avid, with avidity corresponding to the grade of the tumor.^124^ Therefore, it is an excellent tool in both diagnosis and therapy. PSMA‐labeled radiotracers are combined with cross‐sectional imaging and are an excellent tool in baseline staging. Early interest based on ^11^C‐choline, ^18^F‐choline, or amino acid metabolism (eg, ^18^F‐FACBC) has been largely succeeded by small polypeptide ligands to PSMA.^125^ PSMA‐positron emission tomography (PET) scans have greater sensitivity and specificity compared with conventional imaging (CT and bone scan), a lower radiation dose, and reduced scan time, although this has not yet been shown to improve clinical outcomes.^126^, ^127^, ^128^ The presence of nodal disease on a baseline PSMA‐PET may predict medium‐term oncologic outcomes.^129^ Whole‐body MRI also has greater sensitivity than conventional imaging and is particularly useful for assessing bone metastases.^130^, ^131^ PSMA‐PET is recommended as baseline staging in all high‐risk patients and can be considered for unfavorable‐intermediate‐risk patients.^76^ A PET scan optimally should be performed before initiating ADT because it may affect detection sensitivities.^113^ The global health economic impact of PSMA scans has not been fully determined.^132^, ^133^

**FIGURE 1:**
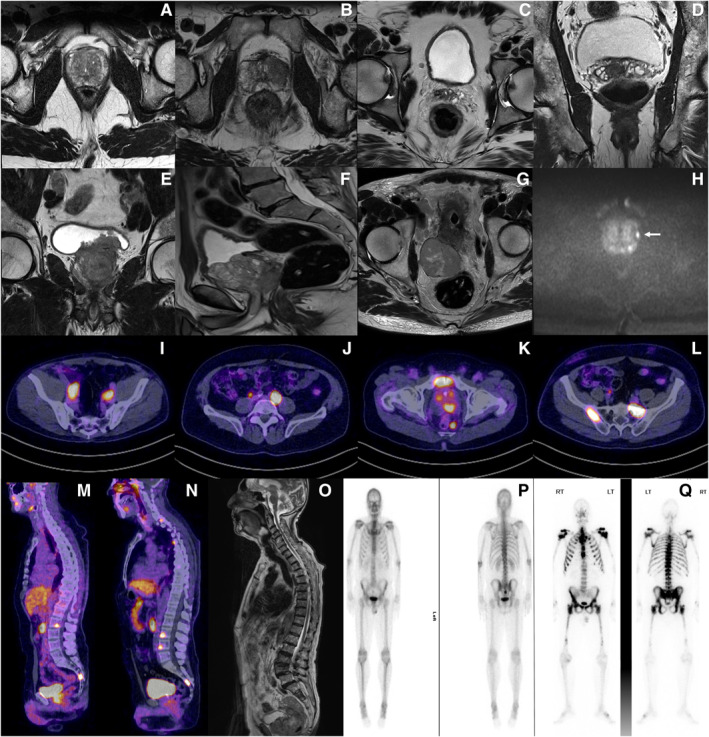
Diagnostic imaging for prostate cancer showing prostate MRI (A‐H), PSMA‐PET (I‐N), whole‐body MRI (O), and bone scans (P, Q). MRI features of organ‐confined disease, (A) T2; capsular bulge and extraprostatic extension, (B) T3a; axial and coronal views of seminal vesicle involvement, (C, D) T3b; adjacent organ involvement, T4, involving (E) urinary bladder, (F) urethra; (G) an exophytic prostatic primary lesion; (H) diffusion‐weighted MRI showing restriction in the left peripheral zone; PSMA‐PET scan showing bilateral pelvic lymph nodes, (I) N1; common iliac lymph node, (J) M1a; mesorectal lymph node, (K) M1a; pelvic bone metastases, (L) M1b; with (M) low‐volume and high‐volume (N) bone metastases; (P) bone scan showing isolated uptake in the sacrum with low‐volume, and (Q) high‐volume bone metastases, *superscan*. Metastatic volume is defined on conventional scans (CT and bone scan) and not PSMA‐PET. CT indicates computed tomography; MRI, magnetic resonance imaging; PET, positron emission tomography; PSMA, prostate‐specific membrane antigen.

Metastatic disease is further classified as high‐volume or low‐volume based on the CHAARTED (Chemohormonal Therapy Versus Androgen Ablation Randomized Trial for Extensive Disease in Prostate Cancer) criteria (ClinicalTrials.gov identifier NCT00309985). High‐volume disease is defined as either four or more bone metastases with one or more outside the vertebral bodies or pelvis, or visceral metastases, or both. This is based on conventional CT and bone scans.^134^ The interpretation of disease burden in light of PET scans is currently unclear. A retrospective study reported similar discrimination based on a PSMA‐directed tumor volume of 40 cm^3^ on a receiver operating characteristic curve in a cohort of 105 patients from three German centers.^135^ Prospective validation is lacking and may be guided by artificial intelligence in the future. PET scans are more sensitive than conventional imaging for nodal and bone staging and are particularly helpful in the diagnosis of small lesions at low serum PSA levels.^136^, ^137^ Diagnostic imaging is represented in Figure 1.

Prostate cancer is currently staged using the 2018 classification (eighth edition) of the American Joint Committee on Cancer (AJCC) Cancer Staging Manual (Table 1).^138^, ^139^ Risk classification is described in Table 2.

**TABLE 1:** Prostate Cancer American Joint Committee on Cancer TNM classification (eighth edition, 2018).^a^

Category	Criteria
Clinical T (cT): Primary tumor	
TX	Primary tumor cannot be assessed
T0	No evidence of primary tumor
T1	Clinically inapparent tumor that is not palpable
T1a	Tumor incidental histologic finding in 5% or less of tissue resected
T1b	Tumor incidental histologic finding in more than 5% of tissue resected
T1c	Tumor identified by needle biopsy in one or both sides, but not palpable
T2	Tumor is palpable and confined within prostate
T2a	Tumor involves one‐half of one side or less
T2b	Tumor involves more than one‐half of one side, but not both sides
T2c	Tumor involves both sides
T3	Extraprostatic tumor that is not fixed or does not invade adjacent structures
T3a	Extraprostatic extension (unilateral or bilateral)
T3b	Tumor invades seminal vesicle(s)
T4	Tumor is fixed or invades adjacent structures other than seminal vesicles, such as external sphincter, rectum, bladder, levator muscles, and/or pelvic wall
Pathologic T (pT): Primary tumor	
T2	Organ confined
T3	Extraprostatic extension
T3a	Extraprostatic extension (unilateral or bilateral) or microscopic invasion of bladder neck
T3b	Tumor invades seminal vesicle(s)
T4	Tumor is fixed or invades adjacent structures other than seminal vesicles, such as external sphincter, rectum, bladder, levator muscles, and/or pelvic wall
N: Regional lymph nodes	
NX	Regional lymph nodes cannot be assessed
N0	No positive regional nodes
N1	Metastases in regional node(s)
M: Distant metastasis	
M0	No distant metastasis
M1	Distant metastasis
M1a	Nonregional lymph node(s)
M1b	Bone(s)
M1c	Other site(s) with or without bone disease
PSA values, ng/mL	Used to assign this category
<10	
≥10 to <20	
<20	
≥20	
Any value	
Histologic grade group	Used to assign this category
1	Gleason score ≤6, Gleason pattern ≤3 + 3
2	Gleason score 7, Gleason pattern 3 + 4
3	Gleason score 7, Gleason pattern 4 + 3
4	Gleason score 8, Gleason pattern 4 + 4, 3 + 5, 5 + 3
5	Gleason score 9 or 10, Gleason pattern 4 + 5, 5 + 4, or 5 + 5

Abbreviation: PSA, prostate‐specific antigen.

^a^ Used with permission of the American College of Surgeons (Chicago, Illinois). The original source for this information is the American Joint Committee on Cancer’s AJCC Cancer Staging System (2023).

**TABLE 2:** Prognostic risk groups for prostate cancer.^a^

Risk group	PSA, ng/mL	Gleason score	Clinical stage
Five‐tier classification according to NCCN			
Very low risk	<10	≤6 (grade group 1)	T1c
Low risk	<10	≤6 (grade group 1)	T1c–T2a
Intermediate risk	10–20	7 (grade group 2–3)	T2b–T2c
High risk	>20	8–10 (grade group 4–5)	T3–T4
Very high risk	>40	8–10 (grade group 4–5)	T3–T4
Three‐tier risk classification according to AUA/ASTRO (Eastham 2022^140^), other proposed three‐tier classifications have been described (D'Amico 1998,^114^ Tward 2024,^115^ European Association of Urology 2024^141^)			
Low risk	<10 and	≤6 (grade group 1) and	T1–T2a
Intermediate risk	10–20 or	7 (grade group 2–3) or	T2b–T2c
High risk	>20 or	8–10 (grade group 4–5) or	≥T3a

Abbreviations: ASTRO, American Society for Radiation Oncology; AUA, American Urological Association; PSA, prostate‐specific antigen.

^a^ Additional clinical and pathologic features are also considered in National Comprehensive Cancer Network (NCCN) risk stratification. Adapted with permission from the NCCN Clinical Practice Guidelines in Oncology (NCCN Guidelines®) for Prostate Cancer V.1.2025. © 2024 National Comprehensive Cancer Network, Inc. All rights reserved. The NCCN Guidelines® and illustrations herein may not be reproduced in any form for any purpose without the express written permission of NCCN. To view the most recent and complete version of the NCCN Guidelines, go online to NCCN.org. The NCCN Guidelines are a work in progress that may be refined as often as new significant data become available.

## TREATMENT

### Curative (radical‐intent) treatment

**FIGURE 2:**
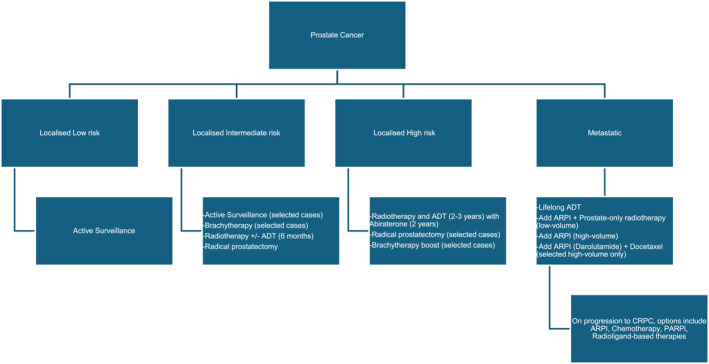
Overview of treatment options for prostate cancer. +/− indicates with or without; ADT, androgen‐deprivation therapy; ARPI, androgen receptor pathway inhibitor; CRPC, castrate‐resistant prostate cancer; PARPi, poly(adenosine diphosphate ribose) polymerase inhibitor.

The overall management of localized prostate cancer is based on risk stratification (Figure 2).^75^, ^113^, ^114^, ^116^, ^117^ When treatment, rather than surveillance, is indicated, individuals should see both a urologic surgeon and a radiation oncologist to discuss the benefits and risks of each suitable treatment modality. Alternatively, for men in whom the cancer is unlikely to become symptomatic or who need treatment during their natural lifespan, *watchful waiting* can be chosen to avoid treatment in those with a short prognosis (<5 years).

#### Active surveillance

Active surveillance refers to a policy of close monitoring with low‐risk or intermediate‐risk disease to defer or avoid curative treatment, balancing cancer control and urinary, bowel, or sexual toxicities. The aim of active surveillance is to detect progression to a higher risk cancer for which radical treatment can be offered.^142^ This approach was established in the 1990s after the indolent natural history of many PSA‐detected prostate cancers was recognized.^143^, ^144^, ^145^, ^146^, ^147^ Early classification systems identifying patients suitable for surveillance were developed.^114^, ^148^

Prospective studies with predefined eligibility criteria in patients with low‐grade disease, based on the studies by Epstein et al.^148^ or D'Amico et al.,^114^ were reported subsequently in the 2000s demonstrating the feasibility of active surveillance. The follow‐up protocol included PSA testing (at three to 12‐month intervals), DRE (six to 12‐month intervals), a confirmatory biopsy within one year, and follow‐up biopsies at intervals of one to three years.^149^, ^150^, ^151^, ^152^, ^153^, ^154^, ^155^ Further evidence came from three clinical trials in the 2010s.^156^, ^157^, ^158^ A meta‐analysis reported only eight prostate cancer‐related deaths and five instances of metastasis during 24,981 person‐years of follow‐up in 7627 men from 26 active surveillance cohorts.^159^

The Scandinavian Prostate Cancer Group SPCG‐4 trial randomized 695 men from Sweden, Finland, and Iceland with localized prostate cancer between 1989 and 1999 (pre‐PSA era) to undergo radical prostatectomy or watchful waiting (not active surveillance). Although, at 30 years of follow‐up, there was a significant relative reduction in overall mortality (26%) and prostate cancer‐specific mortality (48%) in favor of surgery with a mean of 2.2 life‐years gained, subset analyses of the initial analysis demonstrated very low rates of prostate cancer‐specific mortality in men with low‐risk disease (PSA <10 ng/mL and GS <7) and in men older than 65 years, with no significant difference noted between the two arms at 15 years.^156^, ^160^ Most men in this study had advanced disease, and few in the watchful‐waiting arm ever received active treatment.^161^

The American PIVOT trial (Prostate Cancer Intervention Versus Observation Trial) randomized 731 men with localized prostate cancer to radical prostatectomy or watchful waiting between 1994 and 2002 (the era of early PSA testing). At a median follow‐up of 10 years, there was no significant difference in overall survival (OS) or prostate cancer‐specific mortality for men with PSA <10 ng/mL.^157^ However, on extended follow‐up, one life‐year was gained with surgery.^162^

The ProtecT trial from the United Kingdom (International Randomized Controlled Trial Number [ISRCTN] 20141297; ClinicalTrials.gov identifier NCT02044172) randomized 1643 men with screen‐detected prostate cancer to radical prostatectomy, radical radiotherapy, or active monitoring between 1999 and 2009. The proportion of men with intermediate‐risk and high‐risk disease according to contemporary risk stratification was 34% (D'Amico risk classification) and 29% (Cancer of the Prostate Risk Assessment [CAPRA] score, Cambridge Prognostic Group).^163^ Patients in the active monitoring arm underwent PSA testing every three months in the first year and every 6–12 months thereafter without the need for protocol‐defined re‐biopsies; MRI was not routinely available in this era. Patients were considered for definitive treatment if their PSA levels rose by greater than 50% in one year. There was no difference in overall mortality or prostate cancer‐specific mortality on follow‐up at 10 and 15 years. A higher rate of metastasis in the active monitoring arm was presumed to be driven by the proportion of intermediate‐risk and high‐risk cancers.^158^, ^163^ Urinary and erectile functions were better in the monitoring arm.^164^, ^165^

The approach to active surveillance is evolving, and data show that patients in GG 1^100^ have a metastasis risk from <1‐2% and a <1% rate of prostate cancer mortality at 10–15 years.^166^, ^167^, ^168^ Modern multiparametric MRIs and targeted biopsies have improved detection rates and reduced the diagnosis of clinically insignificant cancers.^87^ This approach allows routine biopsies to be avoided, which is beneficial for the patient.^169^ The use of biopsy tumor volume, such as the number of positive cores or the percentage of core involvement, as an eligibility factor for surveillance is now questionable, especially as biopsy targeting strategy evolves.^170^ A PSA increase alone does not justify intervention.^171^, ^172^, ^173^ A PSA doubling time of <3 years has a weak link with grade progression.^174^ It is uncertain whether MRI‐detected cancers pose the same long‐term oncologic risk as cancers of the same grade diagnosed by systematic biopsy.^175^, ^176^

Currently, active surveillance is the preferred treatment for all individuals with low‐risk prostate cancer with a life expectancy of >10 years, and for favorable intermediate‐risk (either GG 1 with PSA <20 ng/mL and ≤T2, or GG 2 with PSA <10 ng/mL and ≤T2 with low tumor volume on MRI and biopsy). Safe surveillance is achieved by a combination of PSA testing every six months, multiparametric MRI every 12–24 months, consideration of biopsy at 18–24 months, and/or an MRI‐directed re‐biopsy on concerns of progression.^113^, ^141^, ^177^, ^178^ Patient or family anxiety about cancer progression is a valid reason to switch to active treatment.^179^

#### Surgery

Active treatment is recommended for individuals with intermediate and high grade localized prostate cancer and a life expectancy of >10 years. The SPCG‐4 randomized trial has demonstrated a mortality reduction in clinically detected disease. The efficacy of surgery is less clear for those with screen‐detected disease.^160^, ^180^ The usual PSA threshold for surgery is <20 ng/mL, although individuals beyond this threshold can be still considered for surgery if they have localized disease on imaging.^181^, ^182^ Most patients with high‐grade prostate cancer are at a higher risk for metastases, require multimodality therapy, and merit multidisciplinary discussion. Most of those who are considering surgery are aged below 70, although occasionally very healthy individuals older than 70 years can be considered. Overall health status, including comorbidities, and not chronological age, should be taken into the decision‐making process when considering an individual for treatment.^183^, ^184^, ^185^ Relative contraindications are obesity or high anaesthetic risk/severe comorbidity. Oncogeriatric assessment, including comprehensive geriatric assessment and estimation of risk of death from coexisting comorbid conditions, can be useful.^186^, ^187^

Robotic‐assisted prostatectomy is the most common surgical technique, followed by radical retropubic or perineal approach. The use of multiparametric MRI combined with a validated nomogram can help predict extraprostatic extension and plan surgery.^92^, ^188^ Nerve‐sparing prostatectomy can be safely undertaken in most cases, and preserving parasympathetic nerves of the pelvic plexus may spare erectile function.^189^ Improved outcomes after surgery are directly correlated with the volume of procedures performed in the hospital and the experience of the surgeon.^190^, ^191^ There is some evidence to suggest that robotic prostatectomy leads to lower postoperative morbidity and margin‐positive resections than laparoscopic prostatectomy, while accounting for methodological uncertainty.^192^ Functional outcomes are similar between an open or a robotic approach.^193^

Surgical complications are classified as: intraoperative (i.e., blood loss, rectal injury, ureteral injury, obturator nerve injury), early (pain, lymphoceles <3%, thromboembolic events <3%) or late (urinary incontinence, impotence).^192^, ^194^ Most patients report return of continence within 3–6 months of surgery. Incontinence rates after prostatectomy are influenced by incontinence definition, and approximately 50%–70% patients are fully continent (no pad use) at one year after surgery.^195^, ^196^, ^197^ There is no difference in continence rates between robotic or open retropubic procedures.^198^ Potency is reported to be 30%–75% after unilateral or 65%–75% for bilateral nerve‐sparing surgeries, although the rates appear to be lower when measured by patient‐reported outcomes.^196^, ^197^ Bladder neck contracture usually occurs in <5% of patients but can approach 20% in those undergoing subsequent radiotherapy.^192^, ^194^, ^199^

Although pelvic lymph node dissection (PLND) is frequently done for staging purposes, there are no strong data that support an OS advantage, although one trial suggests a metastasis‐free survival (MFS) advantage to extended PLND (HR, 0.82; 95% CI, 0.71–0.93; *p* = .003).^200^ Global surgical practice varies widely. Preoperative nomograms help predict the risk of individual lymph node involvement.^201^, ^202^ The European Association of Urology states that a risk of >7% is an indication to perform extended PLND, which improves staging but increases complications (19.8% vs. 8.2%).^27^, ^203^, ^204^

After surgery, individuals should have PSA checked at six to eight weeks, and the level ideally should be undetectable (<0.1 ng/mL). Up to 5% may have a detectable PSA (>0.1 ng/mL) postoperatively, which indicates a poorer prognosis.^205^, ^206^ Further PSA tests should be at 6‐month intervals for the first 2–5 years and annually thereafter. A definitively rising PSA is an indication for consideration of salvage radiotherapy. A PSMA‐PET scan is recommended for biochemical recurrence once PSA levels are >0.2 ng/mL.^207^

The landmark clinical trials for surgery in prostate cancer are summarized in Table 3.^156^, ^157^, ^158^, ^160^, ^161^, ^163^, ^180^, ^208^

**TABLE 3:** Landmark randomized phase III trials for surgery in localized prostate cancer.

Trial	Population	Intervention	Comparison	End points	Key results	Follow‐up
SPCG‐4 (Bill‐Axelson 2011,^156^ Holmberg 2024,^160^ Bill‐Axelson 2014^161^)	695 men with localized prostate cancer, clinically detected, predominantly low risk to intermediate risk	Radical prostatectomy (RP)	Watchful waiting	OS, prostate cancer‐specific mortality, metastasis	Overall mortality: 26% reduction with RP (RR, 0.74; 95% CI, 0.64–0.87) Prostate cancer‐specific mortality: 48% reduction with RP (RR, 0.52; 95% CI, 0.40–0.67), Mean 2.2 life‐years gained with RP (95% CI, 1.4–2.9 life‐years gained) Distant metastasis: 16.7% absolute reduction at 23 years (RR, 0.54; 95% CI, 0.42–0.70; *p* < .001)	30 years
PIVOT (Wilt 2012, 2020^157^, ^162^)	731 men with localized prostate cancer, predominantly low‐risk/intermediate risk, PSA‐detected, aged <75 years, PSA < 50 ng/mL, life expectancy >10 years	RP	Observation	OS, prostate cancer‐specific mortality	Overall mortality: 5.7% reduction with RP (RR, 0.92; 95% CI, 0.84–1.01) Mean 1 life‐year gained with RP Prostate cancer‐specific mortality: 4% reduction with RP (HR, 0.63; 95% CI, 0.39–1.02; *p* =.06)	22 years; median, 18.6 years
ProtecT (Hamdy 2016, 2023^158^, ^163^)	1643 men with localized prostate cancer, predominantly low risk to intermediate risk, PSA‐detected	RP, radiotherapy (RT)	Active monitoring (AM)	Prostate cancer‐specific mortality, OS, metastasis, disease progression, initiation of ADT	Prostate cancer‐specific mortality: 2.2% in RP, 2.9% in RT, 3.1% in AM (no significant difference across groups; *p* = .53) Metastasis: 4.7% in RP, 5.0% in RT, 9.4% in AM (*p* < .001)	Median, 15 years

Abbreviations: ADT, androgen‐deprivation therapy; CI, confidence interval, OS, overall survival; PFS, progression‐free survival; PSA, prostate‐specific antigen; RR, relative risk; SPGC, Scandinavian Prostate Cancer Group.

#### Radiotherapy

The use of radioisotopes to treat prostate cancer evolved in the early 1900s with the use of interstitial brachytherapy. External‐beam radiotherapy gained prominence only in the second one half of the 20th century with the discovery of telecobalt and has evolved rapidly since. Radiotherapy is an essential treatment for intermediate‐risk and high‐risk, localized prostate cancer. Technically, conformal radiotherapy delivered using a linear accelerator reduces long‐term proctitis and rectal bleeding (37% vs. 56% Radiation Therapy Oncology Group [RTOG] grade ≥1; *p* = .004; 5% vs. 15% RTOG grade ≥2; *p* = .01) compared with conventional radiotherapy without compromising local control.^209^ However, an increased dose of radiotherapy (dose escalation) improves biochemical control with no benefit in survival at a cost of increased late bowel side effects.^210^, ^211^, ^212^, ^213^, ^214^, ^215^, ^216^ The standard of care in terms of radiotherapy technique is intensity‐modulated radiotherapy or volumetric‐modulated arc therapy with image guidance.

Biologically, the behavior of prostate cancer differs from that of other cancers because of intrinsic differences in the rate of growth and repair of DNA damage and thus has higher sensitivity to an increased radiotherapy dose delivered per fraction, also known as *hypofractionation*.^217^, ^218^, ^219^ Therefore, it was logical to determine whether radiotherapy schedules could be shortened to improve patient convenience and efficiency while maintaining high cure rates.

Moderate hypofractionation (between 2.4 grays [Gy] and 3.4 Gy per fraction)^220^ has been studied in both superiority (Regina Elena, Fox Chase, MD Anderson, HYPRO)^221^, ^222^, ^223^, ^224^ and noninferiority (RTOG 0415 [ClinicalTrials.gov identifier NT00331773], ProfiT, and CHHiP [ISRCTN97182923)^225^, ^226^, ^227^ randomized clinical trials compared with conventional fractionation. CHHiP was the largest randomized phase III clinical trial to test moderate hypofractionation in a population of 3216 patients from 71 centers in the United Kingdom, the Republic of Ireland, Switzerland, and New Zealand. The trial randomized individuals with T1b–T3aN0M0 prostate cancer, PSA ≤30 ng/mL, and a maximum GS of 4 + 4 to either conventional fractionation (74 Gy in 37 fractions) or one of two hypofractionated regimens (60 Gy in 20 fractions or 57 Gy in 19 fractions) using a noninferiority design. The hypofractionated regimen of 60 Gy in 20 fractions was identified as noninferior for biochemical control (HR, 0.84; 90% CI, 0.72–0.97), with similar OS and late side effects for both hypofractionated arms. For those aged 75 years and older, the regimen of 57 Gy in 19 fractions achieved similar biochemical failure rates with lower gastrointestinal toxicity.^227^, ^228^, ^229^

After it was demonstrated that moderate hypofractionation was at least as good as longer schedules, the next logical step was to test whether treatment could be further abbreviated. Ultrahypofractionation delivers even higher doses per fraction, usually ≥5 Gy.^220^ This concept was first tested in the randomized HYPO‐RT‐PC trial from the Nordic Cancer Union, the Swedish Cancer Society, and the Swedish Research Council (ISRCTN45905321), which compared 42 Gy in seven fractions with conventional fractionation and concluded equivalence for failure‐free survival, albeit with an increase in short‐term, but not long term, side effects.^230^

Stereotactic ablative radiotherapy or stereotactic body radiotherapy (SBRT) is a radiotherapy technique that can deliver ultra‐hypofractionated doses to the tumor accurately while geometrically sparing normal tissues. This can be delivered on a C‐arm linear accelerator, the CyberKnife system (Accuray), or an MR‐linac, which combines magnetic resonance imaging with a linear accelerator. Stereotactic radiotherapy has been shown to be noninferior to longer schedules in the PACE‐B phase III trial (ClinicalTrials.gov identifier NCT01584258) in terms of biochemical or clinical failure for low‐risk to favorable intermediate‐risk disease (GG 2; HR, 0.73; 90% CI, 0.48–1.12; *p* = .004) with a 5‐year control rate of 96%.^231^

The next area of exploration is whether five‐fraction treatments are safe and effective when directed at the prostate and pelvic lymph nodes. This question is currently being addressed in the PACE‐NODES trial (ClinicalTrials.gov identifier NCT05613023), and data from the PRIME trial (ClinicalTrials.gov identifier NCT03561961) indicate low toxicity rates using this approach.^232^, ^233^

MRI‐guided radiotherapy allows daily online adaptation, taking into account changes in tumor and organs‐at‐risk position or shape, with intrafraction motion monitoring, thereby allowing a reduction in margins and potentially reducing toxicities.^234^, ^235^, ^236^ MRI‐guided radiotherapy also represents a potential treatment option for prostate re‐irradiation for local, intraprostatic recurrences.^237^ Radiotherapy has also been identified as effective in boosting the intraprostatic lesion(s), with improved cancer control and acceptable toxicities. Level 1 evidence supports its use with conventional fractionation.^238^, ^239^, ^240^, ^241^ A stereotactic boost regimen is well tolerated.^242^ The use of proton‐beam therapy for prostate cancer results in tumor control and quality of life similar to those achieved with photon‐based treatment in early prostate cancer.^243^

The risk of pelvic nodal involvement in prostate cancer is clinically estimated using the Roach formula (% pelvic lymph node risk = 2/3PSA + (GS‐6)10).^244^ Prophylactic pelvic irradiation can address micrometastatic disease in the pelvic nodes, thereby potentially improving biochemical control and survival. The role of prophylactic pelvic radiotherapy in patients with intermediate‐risk to high‐risk disease who are node‐negative on imaging is controversial.^245^, ^246^, ^247^ Elective pelvic irradiation can improve biochemical control and disease‐free survival at a risk of increasing long‐term toxicities and should be discussed particularly in younger, fitter individuals with very high‐risk disease.^248^, ^249^ Ongoing trial results are awaited.^250^, ^251^

After radical radiotherapy, patients should have PSA checked at six months, then at six‐month intervals for five years, and annually thereafter. Overall, biochemical control with prostate radiotherapy at five years varies by risk group but is estimated to be >90% with either moderate hypofractionation or SBRT.^227^, ^231^ Short‐term side effects from radiotherapy are tiredness, urinary and/or bowel symptoms that resolve 4–8 weeks after radiotherapy completion. Moderate (grade ≥2) late urinary or bowel toxicities are noted in 10%–20% of patients at between 12 and 24 months. The risk of a serious (grade ≥3) toxicity at 2 years after SBRT is <1%.^252^ Stereotactic radiotherapy slightly increases the risk of grade ≥2 urinary toxicity at 12–24 months; however, this difference was not seen in patients treated on a CyberKnife.^252^ Emerging biomarkers may identify patients at higher versus lower risk of urinary toxicity after various forms of radiotherapy.^253^

Patients with intermediate‐risk or high‐risk, localized prostate cancer should be encouraged to make an informed choice about their decision to be treated with prostatectomy or radiotherapy. The median life expectancy after prostate cancer treatment is 13.8 years; therefore, functional outcomes are important determinants of long‐term quality of life.^254^ Prostatectomy is more likely to lead to urinary incontinence or erectile dysfunction but fewer bowel symptoms compared with radiotherapy.^164^, ^165^, ^255^ Similar outcomes were observed in a phase III clinical trial and in a larger, prospective UK multicohort study.^196^, ^197^ Individuals with preexisting poor urinary function should be treated with moderately hypofractionated radiotherapy rather than SBRT.^196^, ^255^ Radiotherapy, particularly external‐beam radiotherapy, entails a small but significant risk of a second primary bladder or colorectal cancer (less than one in 250 individuals).^256^, ^257^

#### Androgen deprivation therapy alongside curative therapy

Androgen deprivation therapy (ADT) reduces serum testosterone, which inhibits cancer growth and reduces PSA. Earlier published clinical trials established that the addition of ADT to radiotherapy in high‐risk, localized prostate cancer improves OS, with long‐course ADT (≥2 years) being superior to short‐course ADT (6 months).^258^, ^259^, ^260^, ^261^, ^262^ A reduced duration of 18 months of androgen suppression was noninferior for survival with an improved quality of life, although not all men in the three‐year arm received the full course.^263^ A meta‐analysis has demonstrated that the addition of ADT to radiotherapy improves MFS (absolute difference, 8.3%; HR, 0.85; 95% CI, 0.79–0.92) and OS (absolute difference, 7.2%; HR, 0.87; 95% CI, 0.8–0.95) at 12 years.^264^, ^265^ Dose‐escalated radiotherapy combined with ADT improves biochemical recurrence‐free survival but not MFS or OS.^266^ Currently, the recommended duration of ADT is four to six months for intermediate‐risk patients and between 18‐36 months for high‐risk patients. There are some data indicating that, with extremely dose‐escalated radiotherapy, a duration shorter than 24 months may be sufficient.^267^ When using short‐duration ADT (four to six months), sequencing may be important, with one analysis suggesting improved outcomes with concurrent/adjuvant versus neoadjuvant/concurrent sequencing in the context of prostate‐only radiotherapy.^268^ Patients with intermediate‐risk disease and Gleason 3 + 4 disease or less may be treated with radiotherapy alone.^231^

In those receiving postoperative prostate bed radiotherapy, the addition of two years of antiandrogen therapy significantly improved MFS (absolute difference; 8.5% at 12 years; *p* = .005) and OS (absolute difference, 5% at 10 years; HR, 0.77; 95% CI, 0.59–0.99; *p* = .04) compared with placebo.^269^ The RADICALS‐HD trial (ClinicalTrials.gov identifier NCT00541047) has established that long‐course ADT (24 months) improves MFS compared with short‐course ADT (six months; HR, 0.773; 95% CI, 0.612–0.975; *p* = .029).^270^ No definite OS benefit was observed.^271^ There is contradictory evidence regarding the use of six months of ADT, with some trials reporting a benefit in MFS or progression‐free survival (PFS),^272^, ^273^ whereas others have reported none,^274^ so it may be offered based on a risk–benefit discussion with the individual. The three‐way comparison of the RADICALS‐HD trial (none, short‐course, long‐course) failed to detect an MFS benefit.^275^

The time to testosterone recovery depends on the age of the individual, baseline testosterone, and duration of ADT prescribed.^276^ The oral ADT relugolix achieves rapid, sustained testosterone suppression and allows for earlier testosterone recovery upon discontinuation, and it may reduce the risk of major cardiovascular adverse events.^277^, ^278^ The parenteral gonadotropin‐releasing hormone (GnRH) agonist degarelix has a similar cardiac profile compared with a GnRH agonist like leuprolide.^279^

#### Brachytherapy

Brachytherapy using low‐dose‐rate permanent seeds or high‐dose‐rate temporary sources, either alone or in combination with external‐beam radiotherapy, is an option for carefully selected patients who have intermediate‐risk or high‐risk disease. Contraindications for the procedure are: a large transurethral resection defect, poor lower urinary tract symptoms (peak urinary flow rate <10 cm^3^ per second, and postvoid residual volume >100 cm^3^), very large prostate gland, preexisting fistula, absence of a rectum, ataxia telangiectasia, or any comorbidity that precludes anesthesia. The biochemical control rate at five years is >85%, 69%–97%, and 63%–80%, respectively, for low‐risk, intermediate‐risk, and high‐risk disease. The rate of grade 3–4 side effects is typically <5%, although some series indicate significantly higher rates of genitourinary toxicity. The rates of bowel side effects from brachytherapy alone are low.^280^, ^281^, ^282^

The addition of a brachytherapy boost after external‐beam radiotherapy improves biochemical control without affecting OS, but it does increase the risk of toxicity.^283^, ^284^, ^285^, ^286^

#### Radiotherapy after prostatectomy

Prognostic scoring and risk‐stratification models can help stratify risk and outcomes after prostatectomy.^118^, ^287^ Radiotherapy could be delivered adjuvantly (routinely after prostatectomy) or as salvage (for a rising PSA). Early salvage radiotherapy has been proposed to improve all‐cause mortality in individuals with high‐risk factors postprostatectomy.^288^, ^289^, ^290^ Although early trials of adjuvant radiotherapy suggested a benefit,^291^, ^292^, ^293^ more recent trials and a meta‐analysis support the use of observation and early salvage radiotherapy, given adjuvant radiotherapy has no proven benefit in terms of biochemical PFS but increased urinary and bowel toxicity.^272^, ^294^, ^295^, ^296^, ^297^ Outcomes are favorable if radiotherapy is initiated at a PSA level <0.5 ng/mL, but treatment should be initiated as early as possible.^298^ Early salvage radiotherapy is the standard of care, and the decision to treat can be offered at a PSA of 0.2 ng/mL or with two or three consecutive PSA rises after surgery. The addition of pelvic radiotherapy improves freedom from progression when combined with prostate bed radiotherapy and short‐term ADT.^273^ Radiotherapy dose escalation from 64 to 70 Gy did not improve outcomes.^299^

#### Focal therapy

There is insufficient evidence at this time to recommend minimally invasive focal therapy, e.g., high‐intensity focused ultrasound (HIFU) or cryotherapy, for the treatment of localized prostate cancer, particularly because of the need for salvage second‐line treatments and the lack of randomized clinical data, and it should be offered only within a clinical trial or a prospective registry.^76^, ^300^, ^301^, ^302^

#### Systemic treatment for nonmetastatic disease

Abiraterone given for nonmetastatic, high‐risk disease (defined by N1 status or at least two of T3/T4, PSA >40 ng/mL, or GS 8–10) significantly improves MFS (absolute benefit, 13% at 6 years; HR, 0.53; 95% CI, 0.44–0.64; *p* < .0001) and OS, (HR, 0.60; 95% CI. 0.48–0.73; *p* < .0001), as evidenced from a meta‐analysis, and should be recommended for a duration of two years alongside three years of ADT and radiotherapy to the prostate.^303^ Docetaxel chemotherapy given as neoadjuvant treatment in high‐risk, localized prostate cancer can help improve relapse‐free survival but does not have a benefit when given in the adjuvant setting after radical prostatectomy or radical radiotherapy.^304^, ^305^, ^306^, ^307^, ^308^, ^309^, ^310^ Therefore, it is not used often, especially given the introduction of novel anti‐androgens, and is not recommended in localized disease. Clinical trials with apalutamide and enzalutamide are ongoing.^311^, ^312^

### Life‐prolonging treatment

#### Relapse after radical treatment

The PSA level that defines biochemical failure depends on the primary treatment. After radical prostatectomy, the PSA level that best predicts metastases is >0.4 ng/mL,^313^ but the most widely accepted definition of biochemical relapse is PSA >0.2 ng/mL or two consecutive rises.^76^ After radiotherapy, the definition of PSA failure is an increase >2 ng/mL above nadir (the lowest PSA after treatment).^314^ The yield of conventional imaging is low in asymptomatic patients. The probability of a positive result on a bone scan is <5% at a PSA level <7 ng/mL.^141^, ^315^ Therefore, conventional imaging is not recommended for restaging. PSMA‐PET CT has higher sensitivity, specificity, positive predictive value, and rates of detection for failures in the prostate bed, pelvic lymph nodes, and the whole body.^207^, ^316^

A positive scan depends on the PSA level: 33% at <0.2 ng/mL, 45% at 0.2–0.49 ng/mL, 59% at 0.5–0.99 ng/mL, 75% at 1.0–1.99 ng/mL, and 95% at 2 ng/mL.^207^ This can help localize the site of recurrence/metastases and lead to a change in the proposed plan of treatment.^317^

The natural history of relapsed disease is long, and life expectancy and quality of life are important parameters to consider when deciding whether to pursue local treatment.^318^ Indeed, while biochemical relapse‐based end points are prognostic, these are not surrogate end points for OS.^319^ Local treatments include salvage prostatectomy, cryoablation, high‐intensity focused ultrasound, re‐irradiation with external‐beam or brachytherapy, which can provide temporary biochemical control in most patients but have important morbidity considerations.^320^, ^321^ For this reason, local recurrence after radiotherapy should be confirmed by imaging (MRI and/or PET PSMA) and biopsy if imaging is suspicious for local recurrence. ADT is usually initiated on development of symptoms or metastases or with a PSA doubling time less than six months.^322^ Delayed and intermittent ADT is preferred for most patients because early ADT does not improve survival.^323^, ^324^ Enzalutamide in combination with ADT improves MFS compared with ADT alone for high‐risk biochemical recurrences with a PSA doubling time less than nine months, whereas enzalutamide monotherapy may be an option for those struggling with adverse effects of ADT.^325^

#### Radiotherapy for metastatic disease

Radiotherapy has been studied in the context of metastatic disease and has been shown to improve OS in low‐volume disease when added to standard‐of‐care treatment, translating to an expected benefit of more than two years (HR, 0.64; 95% CI, 0.52–0.79; *p* < .001).^326^, ^327^, ^328^, ^329^ The benefit is even greater for those with only nonregional lymph nodes (M1a) or three or fewer bone metastases (HR for OS, 0.62; 95% CI, 0.46–0.83; HR for failure‐free survival, 0.57; 95% CI, 0.47–0.70).^330^ In high‐volume disease, it can reduce the incidence of complications like obstructive uropathy.^331^ The PEACE‐1 trial (ClinicalTrials.gov identifier NCT01957436) demonstrated that adding radiotherapy to standard‐of‐care treatment (with abiraterone) improves radiographic PFS in men with low‐volume disease, reduces serious genitourinary events, and prolongs time to castration‐resistance regardless of disease volume.^332^

Oligometastatic prostate cancer is usually defined as having up to five metastatic lesions and is further subclassified on the basis of the time interval between appearance of metastases as metachronous or synchronous.^333^, ^334^ Metastasis‐directed therapy can reduce the rate of biochemical recurrence, increase the time to subsequent therapy, and improve PFS^335^, ^336^, ^337^, ^338^, ^339^; however, an OS benefit has not yet been demonstrated in prostate cancer. The use of stereotactic radiotherapy has been shown to improve biochemical control and PFS in a randomized phase II trial of patients starting first‐line therapy for castration‐resistant disease.^340^ Whole‐pelvic nodal radiotherapy improves biochemical and regional control compared with metastasis‐directed therapy for oligorecurrent pelvic nodal disease and should be considered the standard of care for pelvic nodal oligorecurrence.^341^, ^342^

#### Metastatic hormone‐sensitive prostate cancer

Approximately 5%–15% of all prostate cancers are metastatic (stage IV) at presentation, whereas this proportion can increase to 20%–25% or more in countries with limited access to health care.^343^, ^344^, ^345^ Reducing circulating testosterone is the backbone of metastatic prostate cancer treatment.^346^, ^347^ Metastatic disease is initially responsive to testosterone suppression (hormone‐sensitive prostate cancer [HSPC]or castration‐sensitive prostate cancer) but eventually progresses to a hormone‐resistant phase (castration‐resistance).

Androgen deprivation can be achieved by surgical (bilateral orchidectomy) or medical castration. Hormonal agents include GnRH agonists (leuprolide, goserelin, triptorelin, buserelin) or GnRH antagonists (degarelix, relugolix). Androgen receptor pathway inhibitors (ARPIs) act on the androgen receptor pathways by either competing with androgens for binding to androgen receptors or by deeply suppressing androgen production. ARPIs are classified as first‐generation (flutamide, bicalutamide, nilutamide) or second‐generation (apalutamide, enzalutamide, darolutamide). Second‐generation ARPIs are considered more potent. Darolutamide is the only ARPI that does not cross the blood–brain barrier, thereby helping to reduce neurologic side effects.^348^ Abiraterone is a potent CYP17A1 inhibitor that blocks the biosynthesis of androgens in the adrenal glands, testis, and the tumor itself.^349^ Key adverse effects are summarized in Table 4.

**TABLE 4:** Adverse effects of androgen‐deprivation therapy and androgen receptor pathway inhibitors.

Therapy	Common adverse effects	Serious or notable adverse effects	Notes
Androgen‐deprivation therapy	Hot flashes, fatigue, gynecomastia, weight gain, loss of libido, muscle wasting, mood changes	Osteoporosis, cardiovascular risk, metabolic syndrome, risk of diabetes	Increases the risk of fractures because of bone density loss and may contribute to cognitive decline in long‐term use.
Abiraterone	Hypertension, hypokalemia, fluid retention, liver enzyme elevation	Adrenal insufficiency, hepatotoxicity	Is co‐administered with steroids to manage syndrome of secondary mineralocorticoid excess.
Enzalutamide	Fatigue, hot flashes, musculoskeletal pain, hypertension	Seizure risk, cognitive effects, cardiovascular events	Can cross the blood–brain barrier, potentially causing mild cognitive impairment and increasing fall risk, especially in older patients.
Apalutamide	Fatigue, rash (common), falls, joint pain	Fractures, cardiovascular risk, rare but severe rash, hypothyroidism	Rash is a notable side effect and generally is manageable but occasionally severe enough to warrant dose adjustment.
Darolutamide	Fatigue, pain in extremities, hypertension	Minimal blood–brain barrier penetration, few cognitive or seizure‐related side effects	The structure limits central nervous system penetration.

With ADT alone, individuals with high‐grade disease have a median failure‐free survival of 11 months and a median OS of about 42 months.^350^ Maximal androgen blockade, defined as the addition of a first‐generation anti‐androgen like bicalutamide to luteinizing hormone‐releasing hormone (LHRH) analogues, shows a modest reduction in the risk of death with an increased risk of adverse effects.^351^ The addition of docetaxel^134^, ^306^, ^307^, ^352^, ^353^, ^354^ or ARPIs like abiraterone,^355^, ^356^, ^357^, ^358^, ^359^ enzalutamide,^360^, ^361^, ^362^, ^363^ or apalutamide,^364^, ^365^ or with ADT significantly improve OS in metastatic HSPC (mHSPC). Darolutamide in combination with ADT improves radiologic PFS; mature OS data are awaited.^366^ The quantum of benefit with docetaxel in reducing the risk of death is the highest with high‐volume synchronous metastasis.^367^ Table 5 summarizes the results of selected landmark phase III clinical trials. These trials excluded patients with poor performance status or significant comorbidities, and the average age was younger than that seen in a real‐world setting. It is known that ARPIs can increase all‐grade cardiovascular events, including increases in hypertension, arrythmia, or cardiac death.^368^ Patients with de novo mHSPC comprised the majority of those in the phase III trials, and caution should also be exercised when extrapolating these data for patients who relapse after primary treatment. All patients who are sufficiently fit should be offered dual therapy with ADT plus another agent; where available, we suggest this should be an ARPI rather than docetaxel. The added value of an ARPI to ADT + docetaxel was proven in two phase III trials with improved MFS and OS, whereas the added value of docetaxel to ADT + ARPI remains unknown.

**TABLE 5:** Selected trials of combination therapy for hormone‐sensitive metastatic prostate cancer.

Trial	Arms (no. of patients)	Primary end point	PFS, months	OS, months	PFS	OS
GETUG AFU15 (Gravis 2013^352^)	ADT + docetaxel (*n* = 192) vs. ADT (*n* = 193)	OS	22.9 vs. 12.9	62.1 vs. 48.6	HR, 0.67 (*p* < .001)	HR, 0.88 (*p* = .3)
CHAARTED (Sweeney 2015,^134^ Kyriakopoulos 2018^354^)	ADT + docetaxel (*n* = 397) vs. ADT (*n* = 393)	OS	20.2 vs. 11.7	57.6 vs. 47.2	HR, 0.61 (*p* < .001)	HR, 0.72 (*p* = .018)
STAMPEDE (docetaxel; James 2016,^306^ Clarke 2019^353^)	ADT + docetaxel (*n* = 724) vs. ADT (*n* = 362)	OS	53.4 vs. 40.7, RMST	63.1 vs. 57.1, RMST	HR, 0.69 (*p* < .001)	HR, 0.81 (*p* = .003)
LATITUDE (Fizazi 2017, 2019^355^, ^359^)	ADT + abiraterone (*n* = 597) vs. ADT (*n* = 602)	rPFS, OS	33 vs. 14.8	53.3 vs. 36.5	HR, 0.47 (*p* < .001)	HR, 0.66 (*p* < .0001)
STAMPEDE (abiraterone; James 2017, 2022^356^, ^357^)	ADT + abiraterone (*n* = 501) vs. ADT (*n* = 502)	OS	62 vs. 47, RMST	66.0 vs. 54.0, RMST	HR, 0.58 (*p* < .0001)	HR, 0.60 (*p* < .0001)
ARCHES (Armstrong 2019, 2022^360^, ^362^)	ADT + enzalutamide (*n* = 574) vs. ADT (*n* = 576)	rPFS	49.8 vs. 38.9	Not reached	HR, 0.63 (*p* < .001)	HR, 0.66 (*p* < .001)
ENZAMET (Davis 2019,^361^ Sweeney 2023^363^)	ADT + enzalutamide (*n* = 563) vs. ADT (*n* = 562)	OS	68 vs. 22 (PSA), 81 vs. 25 (clinical)	Not reached, 10% improvement at 5 years	HR, 0.44 (PSA); HR, 0.45 (clinical; both *p* < .01)	HR, 0.70 (*p* < .0001)
TITAN (Chi 2019, 2021^364^, ^365^)	ADT + apalutamide (*n* = 525) vs. ADT (*n* = 527)	rPFS, OS	Not reached vs. 44	Not reached vs. 52.2	HR, 0.62 (*p* < .0001)	HR, 0.65 (*p* < .0001)
ARANOTE (Saad 2024^366^)	ADT + darolutamide (*n* = 446) vs. ADT (*n* = 223)	rPFS	Not reached vs. 25	Not reached, OS not mature)	HR, 0.54 (*p* < .0001)	HR, 0.81 (*p* = NS)

Abbreviations: ADT, androgen‐deprivation therapy; HR, hazard ratio; NS, nonsignificant; OS, overall survival; PFS, progression‐free survival; PSA, prostate‐specific antigen; RMST, restricted mean survival time (proportional hazards); rPFS, radiographic progression‐free survival.

Triplet or quadruplet therapeutic options have been studied in mHSPC in two important clinical trials. The PEACE‐1 trial, with a 2 × 2 factorial design, randomized 1173 individuals with mHSPC to standard of care (ADT with or with intravenous docetaxel), standard of care plus radiotherapy, standard of care plus abiraterone, or standard of care plus radiotherapy plus abiraterone. At a median follow‐up of 3.5 years, the combination of ADT, docetaxel, and abiraterone improved OS and radiographic PFS (rPFS) compared with ADT and docetaxel alone. The combination of standard of care with abiraterone and radiotherapy produced the best outcomes in terms of rPFS and OS.^369^, ^370^

The ARASENS trial (ClinicalTrials.gov identifier NCT02799602) randomized 1306 individuals with mHSPC to darolutamide or placebo, both in combination with ADT and docetaxel. The addition of darolutamide significantly improved OS (HR, 0.68; 95% CI, 0.57–0.80; *p* < .001).^371^ However, the *standard‐of‐care* arm in the ARASENS trial was ADT + docetaxel, which is no longer considered optimal. A subsequent meta‐analysis demonstrated no OS benefit for triplet therapy in low‐volume disease, and triplet therapy is not superior to an ARPI doublet for high‐volume disease.^372^ At this time, the combination of ADT, ARPI, and prostate radiotherapy remains a standard of care for low‐volume disease, and triplet or quadruplets with docetaxel are best reserved for de‐novo, high‐burden mHSPC, although optimal patient selection is still a matter of debate.^373^, ^374^ Patients should be advised that there will be a definite quality‐of‐life detriment when adding docetaxel chemotherapy to an ARPI doublet in mHSPC and that, if there is a benefit to having all three agents at diagnosis of metastatic disease, the magnitude of that benefit is not known.^375^

PSMA‐based radioligand therapy has been shown to improve PSA control, delay progression to CRPC, and improve rPFS (HR, 0.58; 95% CI, 0.3–1.0) compared with docetaxel in high‐volume mHSPC in the UpFrontPSMA phase II study (ClinicalTrials.gov NCT04343885).^376^

#### Castration‐resistant prostate cancer

Castration‐resistant prostate cancer is defined as progression of disease with serum testosterone at castrate levels (<50 ng/dl or 1.7 nmol/liter).^315^ Progression can include either multiple PSA rises or radiologic progression.

##### Nonmetastatic castration‐resistant prostate cancer

Nonmetastatic CRPC is identified by the absence of metastatic disease on conventional imaging (CT and bone scan). Nonmetastatic CRPC develops as a result of the use of early and long‐term ADT in nonmetastatic prostate cancer or the initiation of early ADT in patients who have biochemical failure in whom the site of recurrence has not yet been detected.^75^ The latter practice is no longer recommended, hence this scenario will become rarer in the future. Many patients previously classified with nonmetastatic CRPC would now have visible disease on modern imaging. PSMA‐PET/CT is superior to conventional imaging for the detection of metastatic disease.^377^ The use of PSMA‐PET identified the site of disease in 98% of patients previously classified with nonmetastatic CRPC on conventional scans in a retrospective cohort on the basis of the PROMISE (Prostate Cancer Molecular Imaging Standardized Evaluation) criteria: local relapse, 24%; only pelvic disease, 44%; M1 disease, 55%, of whom about 30% had bone or visceral metastases).^378^, ^379^ The use of some novel ARPIs (Table 6) in combination with ADT helps improve MFS and OS in nonmetastatic CRPC.^380^, ^381^, ^382^, ^383^, ^384^, ^385^ Patients with luminal tumors or a high score on the genomic classifier Decipher may achieve more a sustained MFS benefit with the novel ARPI, apalutamide.^386^

**TABLE 6:** Phase III trials of androgen receptor pathway inhibitors for nonmetastatic castration‐resistant prostate cancer.

Trial	Arms (no. of patients)	Primary end point	MFS improvement vs. placebo, months	HR for MFS	OS analysis
PROSPER (Hussain 2018,^380^ Sternberg 2020^381^)	ADT + enzalutamide (*n* = 933) vs. ADT (*n* = 468)	MFS	36.6 vs. 14.7	HR, 0.29 (*p* < .001)	Improved: 67 vs. 56.3 months (HR, 0.73; *p* = .001)
SPARTAN (Smith 2018, 2021^382^, ^383^)	ADT + apalutamide (*n* = 806) vs. ADT (*n* = 401)	MFS	40.5 vs.16.2	HR, 0.28 (*p* < .001)	Improved: 73.9 vs. 59.9 months (HR, 0.78; *p* = .016)
ARAMIS (Fizazi 2019, 2020^384^, ^385^)	ADT + darolutamide (*n* = 955) vs. ADT (*n* = 554)	MFS	40.4 vs. 18.4	HR, 0.41 (*p* < .001)	Improved: 6% at 3 years (HR, 0.69; *p* = .003)

Abbreviations: ADT, androgen‐deprivation therapy; HR, hazard ratio; MFS, metastasis‐free survival; OS, overall survival.

##### Metastatic castration‐resistant prostate cancer

The choice of optimal systemic therapy for metastatic CRPC (mCRPC) depends on multiple factors: age, fitness, volume of disease, previous treatment received for de‐novo or metastatic hormone‐sensitive disease, the presence of DNA‐repair defect, and PSMA expression of the disease. ADT should be continued lifelong because androgen receptor signaling remains critical for prostate cancer cell survival and proliferation even in the castration‐resistant phase.^387^

###### Chemotherapy in CRPC

The first approved systemic chemotherapeutic agent was mitoxantrone, which has been superseded by more effective agents like the first generation taxane, docetaxel (given with low‐dose steroids),^388^, ^389^, ^390^ and the second‐generation, semisynthetic taxane, cabazitaxel.^391^, ^392^ Cabazitaxel and docetaxel have been compared in chemotherapy‐naive patients and are equivalent for OS.^393^ Cabazitaxel improves OS after docetaxel treatment compared with mitoxantrone^391^ or a second‐line ARPI (abiraterone)^394^ and thus is an option for second‐line mCRPC after docetaxel. A reduced‐dose regimen is effective for OS with fewer side effects.^395^ In heavily pretreated patients, carboplatin, either alone or in combination with cabazitaxel, shows modest clinical efficacy and should be reserved for cancers with an aggressive phenotype; both PFS and OS are between six and seven months.^396^, ^397^ Other chemotherapeutic agents like carboplatin/cisplatin and etoposide have shown activity, generally of short duration, for variant histologies (small cell/anaplastic), so called either because of histopathologic evidence of small cell or clinical features (visceral metastases, lytic bone metastases, low PSA).^398^, ^399^, ^400^, ^401^, ^402^

###### Non‐chemotherapy treatments for CRPC

The novel anti‐androgens abiraterone and enzalutamide improve OS in patients who are ARPI‐naïve before or after docetaxel.^403^, ^404^, ^405^, ^406^, ^407^, ^408^, ^409^ Low‐dose abiraterone taken with a low‐fat breakfast achieves similar blood levels to standard‐dose abiraterone taken on an empty stomach because of the pharmacokinetic properties of the drug.^410^ The use of Radium‐223, an alpha emitter, significantly improves OS in those with progressive, bone‐predominant mCRPC and can be recommended irrespective of previous docetaxel use.^411^, ^412^ Radium should be given in conjunction with bisphosphonates or denosumab for bone protection.

For individuals who are ARPI‐naïve in the CRPC setting, the PEACE‐3 (ClinicalTrials.gov identifier NCT02194842) results support the use of Radium‐223 in combination with enzalutamide and bone‐protective agents because the combination significantly improves OS compared with enzalutamide alone (HR, 0.69; 95% CI, 0.52–0.90; *p* = .0031).^413^ For those who have been previously treated with an ARPI, a second ARPI has only modest activity and is not considered standard of care.^414^ Low‐dose dexamethasone can improve PSA and achieve symptomatic responses in some.^415^ Key phase III clinical trials of systemic therapies for mCRPC are listed in Table 7.

**TABLE 7:** Landmark clinical trials of systemic therapies for metastatic castrate‐resistant prostate cancer.

Trial	Arms (no. of patients)	Primary end point	Median OS, months	HR for OS	PFS/rPFS
TAX 327 (Tannock 2004,^388^ Berthold 2008^390^)	Docetaxel three‐weekly (*n* = 335) vs. docetaxel weekly (*n* = 334) vs. mitoxantrone (*n* = 337)	OS	19.2 vs. 17.8 vs. 16.3	HR, 0.83 both docetaxel groups (*p* = .04), OS improved with three‐weekly (*p* = .009) but not weekly (*p* = .36) docetaxel	—
CARD (De Wit 2019^394^)	Cabazitaxel (*n* = 129) vs. abiraterone or enzalutamide (*n* = 126; post‐docetaxel)	ibPFS	13.6 vs. 11.0	HR, 0.64 (*p* = .008)	Improved (4.4 vs. 2.7 months; HR, 0.52; *p* < .001)
TROPIC (De Bono 2010^391^, ^392^)	Cabazitaxel (*n* = 378) vs. mitoxantrone (*n* = 377)	OS	15.1 vs. 12.7	HR, 0.70 (*p* < .0001)	Improved (2.8 vs. 1.4 months; HR, 0.74; *p* < .0001)
PROSELICA (Eisenberger 2017^395^)	Cabazitaxel (20 mg/m^2^; *n* = 598) vs. (25 mg/m^2^; *n* = 602)	Noninferiority of OS (HR, <1.214)	13.4 vs. 14.5	HR, 1.024 (noninferior)	Similar (2.9 vs. 3.5 months; HR, 1.099)
FIRSTANA (Oudard 2017^393^)	Cabazitaxel (20 mg/m^2^; *n* = 389) vs. cabazitaxel (25 mg/m^2^; *n* = 388) vs. docetaxel (*n* = 391)	OS	24.5 vs. 25.2 vs. 24.3	C20 (HR, 1.01; *p* = .997), C25 (HR, 0.97; *p* = .757)	Similar (4.4 vs. 5.1 vs. 5.3 months): C20 (HR, 1.06; *p* = .422), C25 (HR, 0.99; *p* = .804)
AFFIRM (Scher 2012^407^)	Enzalutamide (*n* = 800) vs. placebo (*n* = 399)	OS	18.4 vs. 13.6	HR, 0.63 (*p* < .001)	Improved (8.3 vs. 2.9 months; HR, 0.40; *p* < .001)
PREVAIL (Beer 2014, 2017^408^, ^409^)	Enzalutamide (*n* = 872) vs. placebo (*n* = 845)	OS, rPFS	35.3 vs. 31.3	HR, 0.77 (*p* = .0002)	Improved (20 vs. 5.4 months; HR, 0.32; *p* < .0001)
COU‐AA‐301 (De Bono 2011,^403^ Fizazi 2012^404^)	Abiraterone (*n* = 797) vs. placebo (*n* = 398; post‐docetaxel)	OS	15.8 vs. 11.2	HR, 0.74 (*p* < .0001)	Improved (5.6 vs. 3.6 months; HR, 0.66; *p* < .0001)
COU‐AA‐302 (Ryan 2013, 2015^405^, ^406^)	Abiraterone (*n* = 546) vs. placebo (*n* = 542; pre‐docetaxel)	OS, rPFS	34.7 vs. 30.3	HR, 0.81 (*p* = .0033)	Improved (16.5 vs. 8.3 months; HR, 0.53; *p* < .001)
EORTC‐GUCG 1333/PEACE‐3 (Gillessen 2024^413^)	Enzalutamide + radium‐223 (*n* = 222) vs. enzalutamide (*n* = 224)	rPFS	42.3 vs. 35	HR, 0.69 (*p* = .0031)	Improved (19.4 vs. 16.4 months; HR, 0.69; *p* = .0009)
PROPEL (Clarke 2022,^416^ Saad 2023^417^)	Olaparib + abiraterone (*n* = 399) vs. placebo + abiraterone (*n* = 397)	ibPFS	42.1 vs. 34.7	HR, 0.81 (*p* = .054)	Improved (24.8 vs. 16.6 months; HR, 0.66; *p* < .001)
PROFOUND (De Bono 2020,^418^ Hussain 2020^419^)	Olaparib vs. physician’s choice in HRRm patients (cohort A, BRCA1/2 vs. ATM (*n* = 162 vs. *n* = 83); cohort B, other genes (*n* = 94 vs. *n* = 48)	ibPFS in cohort A	Cohort A, 19.1 vs. 14.7; cohort B, 14.1 vs. 11.5; overall, 17.3 vs. 14	Cohort A: HR, 0.69 (*p* = .02); cohort B: HR, 0.96 (*p* = NS); overall: HR, 0.79 (*p* = NS)	Improved: Cohort A, 7.4 vs. 3.6 months (HR, 0.34; *p* < .001); overall: 5.8 vs. 3.5 months (HR, 0.49; *p* < .001)
TRITON3 (Fizazi 2023,^420^ Bryce 2025^421^)	Rucaparib (*n* = 270) vs. physician’s choice (*n* = 135; patients with HRRm)	ibPFS	BRCA subgroup, 23.2 vs. 21.2; overall, 22.8 vs. 21.7	BRCA subgroup: HR, 0.91 (*p* = NS); overall HR, 0.99 (*p* = NS)	Improved: BRCA subgroup, 11.2 vs. 6.4 months (HR, 0.50; *p* < .001); overall: 10.2 vs. 6.4 months (HR, 0.61; *p* < .001)
TALAPRO2 (Agarwal 2023, 2025^422^, ^423^)	Talazoparib + enzalutamide (*n* = 402) vs. placebo + enzalutamide (*n* = 403)	rPFS	45.8 vs. 37.0	HR, 0.796 (*p* = .0155)	Improved: Not reached vs. 21.9 months (HR, 0.63; *p* < .0001)

Abbreviations: EORTC‐GUCG, European Organization for Research and Treatment of Cancer Genito‐Urinary Cancers Group; HR, hazard ratio; HRRm, homologous recombination repair‐mutant; NS, nonsignificant; OS, overall survival; PFS, progression‐free survival; rPFS, radiographic progression‐free survival.

###### Poly(adenosine diphosphate‐ribose) polymerase (PARP) inhibitors

DNA‐repair defects are identified in up to 20%–30% of patients with mCRPC, such as germline or somatic homologous recombination repair genes (BRCA1, BRCA2) or the DNA‐damage checkpoint activator ATM.^424^, ^425^ Somatic genetic testing should be offered to all patients with newly diagnosed mHSPC and is recommended for all mCRPCs. Germline testing guidelines vary, but testing should be encouraged for those with a strong family history of prostate cancer, especially at a young age, or if at least two family members on the same side of the family have been diagnosed with tumors linked to hereditary cancer‐predisposition syndromes (including breast, ovarian, prostate, and pancreatic cancers).^75^, ^113^, ^141^, ^426^, ^427^

Treatment options for mCRPCs harboring homologous recombination repair gene alterations include a poly(adenosine diphosphate ribose) polymerase (PARP) inhibitor (PARPi) alone (olaparib, rucaparib) or in combination with ARPIs (abiraterone + olaparib, abiraterone + niraparib, enzalutamide + talazoparib).^416^, ^417^, ^418^, ^419^, ^420^, ^421^, ^422^, ^423^, ^428^, ^429^ Somatic genetic testing is strongly recommended before commencing a PARPi.^430^ There is insufficient evidence to recommend a PARPi for patients without the mutations shown to benefit.

###### Radioligand‐based therapies

PSMA‐based radioligands can selectively deliver radiation to PSMA‐positive cells and the surrounding microenvironment. The use of Lutetium‐177 (^177^Lu)–PSMA‐617 improved OS compared with the standard of care in patients who received an ARPI and one or two prior taxanes in the VISION trial (HR, 0.62; 95% CI, 0.52–0.74; *p* < .001; ClinicalTrials.gov identifier NCT03511664),^431^ whereas a radiographic PFS benefit was observed in the PSMAfore trial (ClinicalTrials.gov identifier NCT04689828) in taxane‐naïve individuals pretreated with an ARPI compared with a second ARPI (HR, 0.49; 95% CI, 0.39–0.61) with fewer adverse effects.^432^ The SPLASH trial (ClinicalTrials.gov identifier NCT04647526) demonstrated improved rPFS with ^177^Lu‐PNT2002 compared with a change of ARPI (HR, 0.71; 95% CI, 0.55–0.92; *p* = .0088) in chemotherapy‐naïve, ARPI‐pretreated individuals. The crossover rate in that trial was 85%.^433^

**FIGURE 3:**
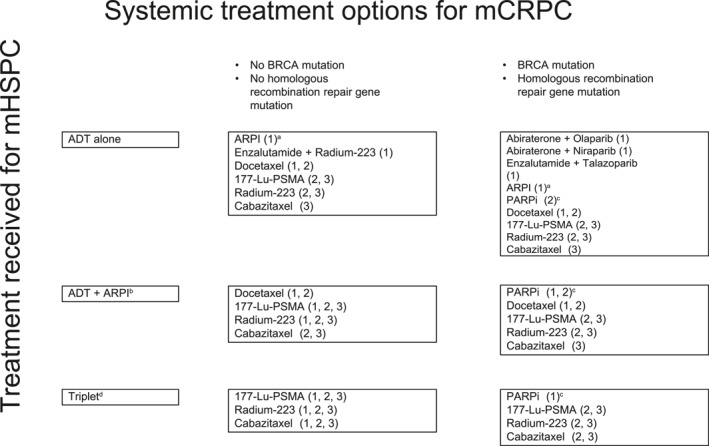
Systemic treatment options for metastatic CRPC. ^a^ARPI in mCRPC, abiraterone, enzalutamide; ^b^ARPI in mHSPC, apalutamide, enzalutamide, abiraterone; ^c^PARPi, olaparib, rucaparib monotherapy approved after previous treatment with an ARPI; ^d^triplet, ADT + docetaxel + darolutamide. The line of treatment in mCRPC is indicated in parentheses. ^177^Lu–PSMA indicates Lutetium‐177 prostate‐specific membrane antigen; ADT, androgen‐deprivation therapy; ARPI, androgen receptor pathway inhibitor; mCRPC, metastatic castrate‐resistant prostate cancer; mHSPC, metastatic hormone‐sensitive prostate cancer; PARPi, poly(adenosine diphosphate ribose) polymerase inhibitor.

There is no agreed consensus regarding the ideal sequencing of these agents. A suggested flowchart is represented in Figure 3. There is insufficient evidence to recommend the routine use of immunotherapy in prostate cancer treatment outside of clinical trials.

**FIGURE 4:**
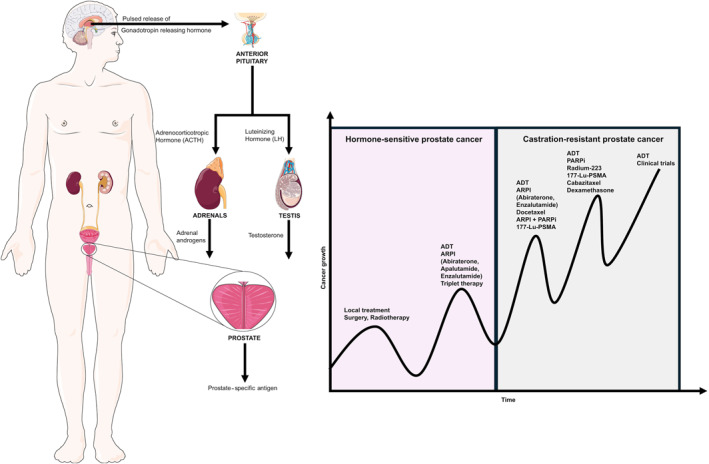
Landscape of the natural history of prostate cancer. ^177^Lu–PSMA indicates Lutetium‐177 prostate‐specific membrane antigen; ADT, androgen‐deprivation therapy; ARPI, androgen receptor pathway inhibitor; PARPi, poly(adenosine diphosphate ribose) polymerase inhibitor. Image adapted from Servier Medical Art (https://smart.servier.com/), licensed under CC BY 4.0 (https://creativecommons.org/licenses/by/4.0/).

Ongoing research focuses on clinical trials of possible therapeutic targets and the identification of novel targets: Akt signaling inhibitors in PTEN loss,^434^, ^435^ PD‐1 inhibitors in microsatellite instability‐high tumors,^436^ selective PARP1 inhibitors,^437^, ^438^ agents that target and inhibit steroid biosynthesis,^439^ small molecules targeting protein degradation,^440^ T‐cell engagers targeting PSMA (human kallikrein 2, STEAP1),^441^, ^442^ and targeting potential markers of neuroendocrine differentiation (DLL‐3, EZH2).^443^, ^444^ The natural history of prostate cancer and its clinical course is represented in Figure 4.

## SURVIVORSHIP AND QUALITY OF LIFE

Over the past four decades, the five‐year survival rate for patients with prostate cancer has increased from 68% to >95%, with that of localized prostate cancer approaching 100%.^445^ More than one in three individuals treated for prostate cancer experience long‐term decrements in their quality of life, with significant impact on their physical, mental, metabolic, and sexual health.^446^, ^447^ These include long‐term toxicities from radical treatment and ongoing adverse effects of treatment for metastatic disease.

The use of lifelong ADT in combination with newer therapeutic options in metastatic prostate cancer can successfully control the disease for many years, thereby leading to a protracted natural course of the disease with features of a *chronic malignancy*. ADT itself is associated with weight gain, reduced muscle mass and bone mineral density, an increased risk of falls and fragility fractures, an increased risk of cardiovascular disease, changes to mood and cognition, depression, loss of sexual function and libido leading to reduced intimacy with their partners, and an increased risk of suicidal ideations.^448^, ^449^, ^450^ Testosterone‐replacement therapy can be offered to individuals who have previously treated prostate cancer with a laboratory diagnosis of testosterone deficiency and a stable PSA after radiotherapy or an undetectable PSA after radical prostatectomy at least 6–12 months after primary treatment, but it should always be part of a shared decision‐making process acknowledging that data are limited and the true risk or safety is unknown. Extreme caution should be exercised in those at high risk for relapse or progression. Those receiving ADT for metastatic disease, either in the hormone‐sensitive or castration‐resistant setting, should not be prescribed testosterone replacement.^451^, ^452^

One in five individuals surviving five years after diagnosis report a fracture on ADT.^453^ The use of additional anti‐androgen therapies with ADT further increases the risk of fractures.^454^ The use of a bisphosphonate reduces fracture risk in patients with M1 status, but not in those with M0 status,.^455^ Zoledronic acid reduces the risk of skeletal complications and delays the time to first bone complication.^456^, ^457^ Denosumab, another bone‐targeting agent, further reduces the chance of a bone complication compared with zoledronate.^458^, ^459^

Individuals on lifelong ADT should receive calcium and Vitamin D supplementation, along with a bisphosphonate or regular dual‐energy x‐ray absorptiometry (DEXA) scans, with treatment as directed. Those who receive ADT for two to three years for high‐risk localized disease should receive calcium/Vitamin D supplementation only. Short‐term ADT for six months does not require additional supplementation. A complete dental evaluation and a completion of invasive dental procedures are recommended before initiating a bone‐targeting agent. Those with a contraindication to a bisphosphonate should be monitored with dual‐energy radiographic absorptiometry scans.

Individuals should be counseled to maintain a healthy weight for their height, avoid high‐calorific food, avoid smoking, and limit alcohol consumption. They should engage in physical activity and weight‐bearing exercises. A structured exercise program leads to improved muscular strength, cardiorespiratory fitness, and functional task performance; reduces fatigue; and helps to improve biomarkers for carbohydrate metabolism, thereby leading to weight loss.^460^, ^461^ Individuals should have access to mental health services and should be offered psychotherapy and pharmacotherapy, as appropriate. Couples should be encouraged to discuss sexual intimacy and should be offered counseling and support services, as needed. Screening for cardiovascular and metabolic diseases should follow usual recommendations (monitoring of blood pressure, lipids, and glucose).^462^ The addition of metformin to ADT significantly improves metabolic parameters like hemoglobin A1c, fasting glucose, total and low‐density‐lipoprotein cholesterol and reduces weight gain and metabolic syndrome.^463^

## FRAIL PATIENTS

Frailty is a multidimensional, complex state of diminished physical reserve resulting in decreased resilience and increased vulnerability to stressors. It is an important consideration in treating elderly patients with prostate cancer.^464^ Estimating life expectancy, differentiating between chronologic and biologic age, and comprehensive geriatric and frailty assessments are recommended to screen and manage patients older than 70 years or those who have >5% weight loss because of chronic illness.^464^, ^465^, ^466^, ^467^, ^468^, ^469^ Management of prostate cancer in such patients should be tailored to their overall health status and personal preferences, with individuals deemed physically fit offered the same treatment options as their chronologically younger counterparts.^184^ A key component in decision making to offer radical‐intent treatment is a life expectancy >10 years; in most developed nations, this is reached at an age between 75 and 80 years in the absence of chronic comorbidities.^185^, ^470^

Tailored treatment decisions with modifications in the usual standards of care are recommended, such as reducing the duration of ADT in those with competing comorbidities. Curative‐intent radiotherapy can be offered to a dose of 57 Gy in 19 fractions in those aged 75 years and older, with biochemical control in >85% but with reduced long‐term late bowel side effects.^229^ Another option is weekly hypofractionated radiotherapy to a dose of 36 Gy in six fractions, extrapolated from the metastatic setting, to treat localized disease, with a five‐year PFS rate >80%.^471^

ARPIs like abiraterone and enzalutamide can also be prescribed safely in older adults and dose‐reduced where needed.^472^, ^473^ The use of Radium‐223 is generally safe for frailer patients, with only minor differences for fracture risk observed beyond second‐line treatment in CRPC compared with other treatment options.^474^

## CONCLUSIONS

Prostate cancer encompasses a wide spectrum of clinical scenarios, ranging from low‐risk disease, in which treatment can only harm and not extend life, to a fatal disease, which still claims too many lives globally. Appropriate risk stratification and individualized treatment are keys to good management. Emerging data and new therapies will continue to refine therapeutic paradigms and improve outcomes for individuals with prostate cancer.

## CONFLICT OF INTEREST STATEMENT

Aidan Adkins reports personal/consulting fees from Novartis; and support for other professional activities from Europa Uomo, Movember Foundation, Prostate Cancer Research, Prostate Cancer UK, and Tackle Prostate Cancer outside the submitted work. Amar Kishan reports grants/contracts from Janssen Biotech Inc. and Point Biopharma; personal/consulting fees from Boston Scientific Corporation, Janssen Biotech, Lantheus, and Varian Medical Systems Inc.; and stock ownership in Viewray Technologies Inc. outside the submitted work. Chris Parker reports personal/consulting fees from Blue Earth Therapeutics, Janssen Pharmaceuticals, and Novartis; and service on a Data and Safety Monitoring board for Telix outside the submitted work. Angela Pathmanathan reports grants/contracts and support for professional activities from Cancer Research UK; and support for other professional activities from Elekta, the Institute of Cancer Research, Janssen Pharmaceuticals, and Prostate Cancer UK outside the submitted work. Alison Reid reports personal/consulting fees/honoraria or travel assistance from Astellas Pharma, AstraZeneca UK Ltd., and Janssen Pharmaceuticals outside the submitted work. Oliver Sartor reports personal/consulting fees from Advanced Accelerator Applications, Amgen, ARTbio, Astellas Pharma, AstraZeneca AB, AstraZeneca Pharmaceuticals LP, Bavarian‐Nordic, Bayer, Clarity Pharmaceuticals, Clovis Oncology Inc., Constellation Pharmaceuticals, Convergent Therapeutics Inc., Blue Earth Diagnostics Ltd., Bristol Myers Squibb, Daiichi Sankyo, Dendreon Pharmaceuticals LLC, EMD Serono, Endocyte, Exelixis, Fusion Pharmaceuticals, Genzyme Corporation, Hengrui Therapeutics AG, Invitae, Isotopen Technologien Meunchen, Janssen Biotech Inc., Janssen Scientific Affairs LLC, Merck, Morphimmune, Moyvant, Myriad Genetic Laboratories Inc., Noria Therapeutics, NorthStar, Novartis, Noxopharm, Pfizer, Point Biopharma, Progenics/Lantheus Medical, Progenics Pharmaceuticals Inc., Sanofi and Genzyme US Companies, Taiho Pharmaceutical, Telix Pharmaceuticals, Tempus, TeneBio, Tessa Therapeutics, and Theranostics outside the submitted work. Nicholas Van As reports grants/contracts, personal/consulting fees, and support for other professional activities from Accuray Inc. and Varian outside the submitted work. Jochen Walz reports grants/contracts from Exact Imaging; and personal/consulting and/or advisory fees/honoraria from A3P, AAA/Novartis, ANNA/C‐TRUS, Astellas Pharma Europe, AstraZeneca, Bayer, Blue Earth Diagnostics, BXTA, Curium US LLC, Intuitive Surgical, Ipsen, Janssen Cilag EAME, Lightpoint Medical, Lucida, Telix, and Veracyte Inc. outside the submitted work. Alison Tree reports grants/contracts from Accuray Inc., Elekta, and Varian; honoraria or travel assistance from Accuray Inc., Bayer, and Janssen Cilag; a gift from Bayer; and travel support from Elekta outside the submitted work. The remaining authors disclosed no conflicts of interest.
